# Towards personalized management of early esophageal adenocarcinoma

**DOI:** 10.1097/MOG.0000000000001030

**Published:** 2024-04-11

**Authors:** Vincent Bos, Man Wai Chan, Roos E. Pouw

**Affiliations:** a Department of Gastroenterology and Hepatology, Amsterdam University Medical Centers; b Cancer Center Amsterdam; c Amsterdam Gastroenterology Endocrinology Metabolism, Amsterdam, The Netherlands

**Keywords:** endoscopic resection, esophageal adenocarcinoma, mucosal cancer, submucosal cancer

## Abstract

**Purpose of review:**

This review aims to discuss recent advancements in the endoscopic management of early esophageal adenocarcinoma (T1 EAC).

**Recent findings:**

Patients with high-risk EAC (defined by the presence of deep submucosal invasion, and/or lymphovascular invasion, and/or poor differentiation) have a higher risk of lymph node metastases than those with low-risk EAC. However, more recent, endoscopically-focused studies report a lower risk of lymph node metastases and distant metastases for high-risk EAC than previously assumed. Instead of referring all high-risk EAC patients for esophagectomy after a radical endoscopic resection, an alternative approach involving regular upper endoscopy with endoscopic ultrasound may allow for detection of intra-luminal recurrence and lymph node metastases at an early and potentially curable stage.

**Summary:**

Endoscopic resection of mucosal and submucosal EAC might prove to be safe and curative for selected cases in the future, when followed by a strict follow-up protocol. Despite the promising results of preliminary studies, there is an ongoing need for personalized strategies and new risk stratification methods to decide on the best management for individual patients with high-risk T1 EAC.

## INTRODUCTION

The incidence of esophageal adenocarcinoma (EAC) is on the rise [1,2]. When diagnosed at an early stage (T1), treatment can often be curative. Traditionally, T1 EAC was treated with radical esophagectomy that included lymph node dissection. In recent decades, therapeutic endoscopic techniques have greatly advanced and now allow for minimally invasive, radical endoscopic resection (ER) of T1 EAC. Esophagectomy has the potential benefit of removing lymph nodes at risk for harboring metastatic disease, however, esophagectomy is associated with considerable mortality rates (0–4%) and much higher complication rates (up to 56%) than ER [3–8]. Consequently, treatment of T1 EAC has largely shifted from surgery to ER [9^▪^,^10^–^12^]. However, due to an assumed risk of lymph node metastases (LNM) of up to 46%, guidelines still recommend consideration of esophagectomy for tumors invading into the submucosal layer (T1b), or if other risk factors such as poor differentiation or lymphovascular invasion (LVI) are found [13^▪▪^]. Interestingly, some recent studies in T1b EAC patients who underwent endoscopic follow-up instead of surgery have demonstrated LNM rates of only 2–31%, rates considerably lower than those suggested by earlier studies [14^▪▪^,^15^,^16^,^17^^▪▪^]. Given these observed discrepancies, the optimal management of high-risk (HR) T1 EAC remains a matter of debate.

In this article, we will discuss the recent developments in endoscopic management of mucosal and submucosal EAC. 

**Box 1:**
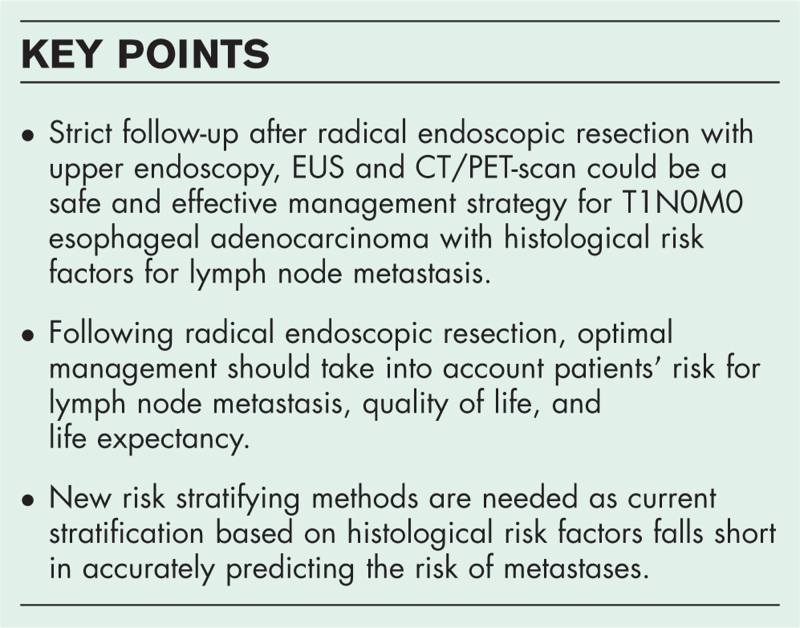
no caption available

## CURRENT PRACTICE

Before deciding on the appropriate management strategy, it is crucial to have adequate tumor staging. ER plays an important role in both the oncological staging and the treatment of T1 EAC. Endoscopic mucosal resection (EMR) and endoscopic submucosal dissection (ESD) are both accepted ER methods. EMR can be either cap-assisted or band-assisted. These are relatively easy-to-master techniques that allow for safe and quick en-bloc resection of lesions ≤20 mm. Larger lesions require piecemeal-resection by EMR. [18,19]. With ESD, the submucosa can be dissected from the muscularis propria after it has been lifted by submucosal injection of fluid. This allows for en-bloc resection of lesions >2 cm, lesions with a bulky intraluminal component, and lesions where there is suspected submucosal invasion or fibrosis, as advised by current guidelines [18,19]. However, ESD has a steeper learning curve and is more time consuming than EMR. Thus far, one randomized controlled trial that included 40 patients assessed the efficacy of ESD versus EMR in BE with a visible lesion ≤3 cm [20]. Although R0 resection (59% vs. 12%, *P* = 0.01) and en-bloc resection rate (100% vs. 15%, *P* < 0.0001) were significantly higher in the ESD group, there was no significant difference between the groups in complete remission after 3 months (94% vs. 94%, *P* = 1.0). Also, the study was powered for R0 resection and not for recurrence, which might have been a clinically more relevant endpoint. Retrospective studies comparing ESD versus EMR in treating EAC have reported varying results, with most finding significantly lower recurrent/residual disease rates after ESD (1% - 23%) versus EMR (13% - 31%) [21–23]. However, the clinical value of these studies is hampered by substantial limitations. The studies were nonrandomized, with differing follow-up (FU) times and skewed distributions in both treatment modality and patient characteristics. Moreover, Abe *et al.* reported solely on Japanese patients, most of whom had short-segment BE or non-BE EAC, features that limit the study's generalizability to Western countries. Whereas en-bloc resection with ESD may indeed be preferable for lesions with suspected submucosal invasion or a bulky intra-luminal component, further research is needed to assess if there is a clinically relevant difference in long-term outcomes between EMR and ESD for flat-type malignant Barrett lesions >2 cm without suspicion for submucosal invasion.

### Current risk stratification

Assessing the risk of LNM is one of the most important factors when deciding on subsequent management after radical ER of T1 EAC. Current risk stratification is based on histopathological tumor characteristics. A T1 EAC is considered to have an increased risk for LNM if there is submucosal invasion, and/or poor tumor differentiation (G3–4), and/or LVI. Given that critical clinical decisions, such as choosing between esophagectomy or esophagus-preserving endoscopic FU depend, among other factors, on the histopathological assessment of these risk factors, accurate staging is crucial. Specimens should be systematically assessed by trained gastrointestinal pathology experts, as histopathological assessment of ER-specimens varies significantly among pathologists [24–26]. Furthermore, it is recommended that specimens should be assessed by a second expert pathologist and should be discussed within a multidisciplinary team (MDT) when HR features are identified that could potentially result in invasive treatment such as surgery or chemoradiation.

### Management of low-risk mucosal esophageal adenocarcinoma

ER has already been established as the preferred treatment of low-risk (LR) T1a EAC (i.e. well/moderate differentiation and no signs of LVI) [13^▪▪^,^14^^▪▪^]. A large prospective cohort study of 1000 patients with LR-T1a EAC treated with ER showed excellent long-term remission rates (93.8%), with no mortality after 5-year FU and a significantly lower EAC-related death than other-cause related death (2 vs. 111) after 10-year FU, with complications in fewer than 2% of patients [27].

### Management of low-risk submucosal esophageal adenocarcinoma

The appropriate management for LR-T1b EACs (i.e. submucosal invasion <500 μm, well/moderate differentiation grade and absence of LVI) is still a topic of discussion due to the scarcity of high-quality, prospective data. Nevertheless, there is a growing trend towards endoscopic FU of LR-T1b EAC after ER [15,16]. Reported LNM rates are around 2%, which is lower than the reported 0–4% mortality associated with esophagectomy, even in expert centers [14^▪▪^,^15^,^17^^▪▪^].

Current guidelines suggest that management of LR-T1b EAC should be performed in expert centers, with a full oncologic staging at baseline with gastroscopy to exclude residual intra-luminal cancer, endoscopic ultrasound (EUS) to assess for LNM, and (PET-)CT to exclude distant metastases. If there are no signs of metastases (T1N0M0), strict endoscopic FU can be considered with gastroscopy and EUS performed at regular intervals. EUS has been proven to be an accurate method for detecting LNM with a reported sensitivity varying between 75% - 100%, exceeding the accuracy of other modalities such as (PET-)CT or MRI [28–31]. When combining the EUS with fine needle aspiration (EUS-FNA) of suspicious lymph nodes, there is an even higher sensitivity of 90–99% for detecting LNM [28,32]. Although current data suggests that strict endoscopic FU (without surgery or chemoradiotherapy) after radical ER of LR-T1b EAC is justified, it must be acknowledged that guidelines are based on low quality evidence and expert opinion.

### Management of high-risk mucosal and submucosal esophageal adenocarcinoma

ER for HR-T1 EAC's, defined as T1a lesions with poor differentiation and/or LVI, and T1b lesions with deep submucosal invasion (≥ 500um) and/or poor differentiation and/or LVI, is currently considered noncurative. Both ESGE and ASGE guidelines recommend that the decision for endoscopic FU or additional treatments, including surgery and/or chemoradiotherapy, should be made after MDT-discussion [13^▪▪^,^14^^▪▪^]. In contrast, the National Institute for Health and Care Excellence (NICE) guideline recommends esophagectomy for HR-T1b EAC, and recommends radiotherapy alone or in combination with chemotherapy when patients are unfit for surgery [33]. However, all guidelines on the management of HR-T1 EACs primarily rely on low-quality data, consisting mostly of older, retrospective studies and surgical series, and expert opinion, underscoring the current scarcity of high-quality evidence in this domain [13^▪▪^,^14^^▪▪^].

A recent retrospective cohort study evaluated the outcomes of patients who were endoscopically managed after radical ER of HR-T1 EAC without metastases at baseline [17^▪▪^]. The study included 120 Dutch patients who underwent endoscopic FU after ER between 2008 and 2019. Patients were categorized in 3 subgroups: HR-T1a (25/120; 21%), LR-T1b (55/120; 46%) and HR-T1b (40/120; 33%) EAC. During a median FU of 35 months, 5/25 (20%) of the HR-T1a patients developed metastases. Among the HR-T1b patients, 3/40 (8%) patients developed metastases during a median FU of 23 months. Median time to metastases in both HR-groups was 24–31 months. EAC related mortality was lower than other-cause mortality (5.8% vs. 13%).

Another recent retrospective cohort study also found a relatively high metastatic rate in patients with HR-T1a [34]. A total of 188 patients who underwent endoscopic FU after successful ER for T1a EAC without baseline metastases between 1996 and 2022 at 3 US centers was included; 143 (76%) patients had LR-T1a EAC and 45 (24%) had HR-T1a. LNM and/or distant metastases occurred in 7/188 (4%) patients at a median FU of 7 months, with a significantly higher (*P* = 0.02) proportion of 5/45 (11%) in the HR-T1a group than the 2/143 (1%) in the LR-T1a group. EAC-related mortality did not differ significantly from any-cause mortality, and mortality between groups did also not differ significantly (7% vs. 2%, *P* = 0.13). However, the study was underpowered for clinical outcomes, and its retrospective nature introduces the possibility of selection bias. Furthermore, FU strategies were not performed according to a standardized FU-protocol.

One research group tried to develop a risk calculator for LNM in patients with T1b EAC, based on retrospectively collected data [35]. They identified all Dutch patients who underwent surgical or endoscopic treatment for T1b EAC from 1989 to 2016 in the Dutch Cancer Registry. All histopathological samples were reviewed to confirm R0 resections. The study included 248 patients, of whom 166 (67%) underwent primary surgery and 82 (33%) underwent ER; among the latter patients, 49 (60%) received additional surgery. After a median FU of 5.5 years, 78/248 (31%) patients developed LNM and/or distant metastasis. The predicted 5-year metastatic (LNM and/or distant) risk was higher for HR-T1b (7–70%) than for LR-T1b EAC (6–16%) (*c*-statistic 0.81, 95% CI 0.75–0.86). However, it is important to acknowledge that the findings were primarily based on data from surgical specimens and the model has not been externally validated for patients treated with ER. Additionally, due to the study's retrospective nature with missing data and variations in initial management, type of surgery, and FU-protocols, there is increased risk of selection, information and lead time bias.

Despite the need for prospective data, the risk of LNM in HR-T1 EACs in the aforementioned studies underscores the critical importance of stringent endoscopic FU after. This FU is crucial for early detection of LNM or intraluminal recurrence at a potentially curable stage.

### Quality of life during endoscopic follow-up

Besides clinical parameters (i.e., recurrence and LNM rates) on which current recommended FU-intervals are based, it is also important to take a patient's health-related quality of life (HRQoL) into account. Currently, data on HRQoL in endoscopically-managed patients with T1 EAC is scarce. A recent review of 6 studies reporting on short-term (i.e., 12 months) HRQoL effects in patients treated for BE-dysplasia and T1 EAC found that undergoing endoscopic treatment could have psychological benefits, while patients undergoing regular surveillance endoscopies could experience increased worry and fear of cancer [36]. However, study populations and management strategies varied greatly. Also, some studies only used general HRQoL-surveys or surveys that were not validated, and the studies were powered for clinical outcomes instead of HRQoL.

Another study on long-term (median FU of 4 years) fear of cancer among 39 endoscopically-treated EAC patients and 28 surgically-treated EAC patients found that worry and fear of cancer recurrence and general anxiety declined over time [37^▪^]. However, endoscopically-treated patients had significantly higher worry of cancer and general anxiety than surgically-treated patients. This might be due to yearly surveillance endoscopies instead of FU only in case of symptoms in the surgically-treated group. However, sample size was small and the clinical relevance of the observed difference remains unknown as no validated cutoffs are available for the questionnaires used. Future studies in this field, also in patients with HR-T1 EAC, are required to better inform patients on what to expect and how their quality of life may be influenced by different treatment options.

## TOWARDS PERSONALIZED MANAGEMENT

To assess the safety of ER of T1b EAC followed by a watchful waiting strategy as an alternative to esophagectomy, the PREFER trial (NCT03222635) was initiated. In this prospective, international, multicenter trial, patients treated with ER for HR-T1b EAC undergo baseline oncological staging with endoscopy, EUS and (PET)CT to exclude lymph node or distant metastases. Patients found to be T1N0M0 provide informed consent for strict FU after radical ER of T1b EACs, consisting of regular upper endoscopies and EUS, with FNA in case of suspicious lymph nodes. During the first 2 years after ER, patients undergo endoscopic surveillance with EUS every 3 months, followed by every 6 months in years 3 and 4, and annually thereafter. Also, a (PET)CT scan is repeated after 1 year of FU. By following a strict FU-protocol, we believe that intraluminal recurrence and/or LNM will be identified at a curable stage. After 5-year FU, the disease-specific mortality/survival and overall survival will be assessed as primary outcome parameters. As secondary parameters, histologically proven LNM or distant metastases, local recurrence eligible for endoscopic therapy, local recurrence requiring surgical therapy, and quality of life will be assessed.

The study also includes HR-T1a EAC in a separate arm, following the same strict FU-protocol. By collecting additional data on HR-T1 EAC, we aim to enhance risk stratification based on histological risk factors, by bridging the current knowledge gap.

### New risk stratification criteria are needed

To decide which patients are good candidates for a strict FU protocol and which would benefit from additional treatment because of their increased risk of LNM, better risk stratification is necessary. Currently, risk stratification is based on infiltration depth, differentiation grade and LVI. However, other risk factors may be relevant as well. Interesting new research directions might include assessment of tumor budding (i.e. single cells or clusters of <5 cancer cells) and the tumor immune micro-environment, which have been identified as prognostic factors in various gastrointestinal tumors [38–42]. In a few retrospective studies, high-grade tumor budding in early EACs has been described as an independent risk factor for LNM and poor prognosis [38,43–45]. Moreover, some studies have also reported a significant correlation of certain immune cell types with LNM and survival rates [46^▪^,47^▪^,^48^]. Although our understanding for EAC is still limited, these results might point towards a possibly valuable direction for further research.

## CONCLUSION

As the incidence of EAC is rising, discussions on how to manage T1 EAC are ongoing. Endoscopic management has been widely accepted for LR-T1a EAC. For patients with HR-T1a and T1b EAC, guidelines suggest that esophagectomy should still be considered. Recent data, however, suggest that, for a select group of patients, strict endoscopic FU with EUS following ER may be a valid approach. However, final results from prospective studies must be awaited. Also, besides adequate histopathological assessment and the development of personalized strategies incorporating HRQoL, there is an ongoing need to explore new LNM risk stratification methods.

## Acknowledgements

*Vincent Bos: Drafting of the manuscript, and final approval of the version to be published.*

*Man Wai Chan: Drafting of the manuscript, and final approval of the version to be published.*

*Roos E. Pouw: Critical revision of the manuscript for important intellectual content, and final approval of the version to be published.*

### Financial support and sponsorship

*No funding was received for the preparation of this manuscript.*

### Conflicts of interest

*Roos E. Pouw is consultant for MicroTech Europe and Medtronic and has received speaker fee from Pentax; Vincent Bos and Man Wai Chan have no conflict of interest to report.*

## REFERENCES AND RECOMMENDED READING

Papers of particular interest, published within the annual period of review, have been highlighted as:

▪ of special interest
▪▪ of outstanding interest
